# MRI-Derived Subcutaneous and Visceral Adipose Tissue Reference Values for Children Aged 6 to Under 18 Years

**DOI:** 10.3389/fnut.2021.757274

**Published:** 2021-10-01

**Authors:** Kacper Marunowski, Dominik Świętoń, Włodzimierz Bzyl, Małgorzata Grzywińska, Mariusz Kaszubowski, Piotr Bandosz, Dmitry Khrichenko, Maciej Piskunowicz

**Affiliations:** ^1^Department of Radiology, Medical University of Gdańsk, Gdańsk, Poland; ^2^Faculty of Mathematics, Physics and Informatics, University of Gdańsk, Gdańsk, Poland; ^3^Department of Human Physiology, Medical University of Gdańsk, Gdańsk, Poland; ^4^Department of Economic Sciences, Faculty of Management and Economics, Institute of Statistics, Gdansk University of Technology, Gdańsk, Poland; ^5^Department of Public Health and Policy, University of Liverpool, Liverpool, United Kingdom; ^6^Department of Prevention and Medical Education, Medical University of Gdańsk, Gdańsk, Poland; ^7^Division of Body Imaging, Department of Radiology, The Children's Hospital of Philadelphia, Philadelphia, PA, United States

**Keywords:** subcutaneous adipose tissue (SAT), visceral adipose tissue (VAT), magnetic resonance imaging, nutritional assessment, age and sex dependent reference values, percentile charts, children

## Abstract

The assessment of body composition in pediatric population is essential for proper nutritional support during hospitalization. However, currently available methods have limitations. This study aims to propose a novel approach for nutrition status assessment and introduce magnetic resonance imaging (MRI)-derived subcutaneous and visceral fat normative reference values. A total of 262 healthy subjects aged from 6 to 18 years underwent MRI examinations and anthropometric measurements. MRI images at the second lumbar vertebrae were used by two radiologists to perform the semi-automatic tissue segmentation. Based on obtained adipose tissue surface areas and body mass index (BMI) scores sex-specific standard percentile curves (3rd, 10th, 25th, 50th, 75th, 90th, 97th) and z-scores were constructed using LMS method. Additionally, 85th and 95th centiles of subcutaneous and visceral adipose tissue were proposed as equivalents of overweight and obesity. Bland-Altman plots revealed an excellent intra-observer reproducibility and inter-observer agreement. In conclusion, our findings demonstrate highly reproducible method and suggest that MRI-derived reference values can be implemented in clinical practice.

## Introduction

Childhood overweight and obesity have been recognized as strong risk factors for the development of cardiovascular disease, diabetes mellitus, depression, and cancer in adulthood (1, 2). Thus, determining body tissue composition, particularly visceral, and subcutaneous adipose tissue compartments can be useful for the assessment of patient risk stratification. A proper development during the growth period requires an appropriate nutritional status, mainly in children with coexisting chronic cardiovascular or oncological diseases (3, 4). In routine clinical practice, the assessment of obesity grade and body fat content is based on anthropometric measures and indexes such as skinfold thickness, body mass index (BMI), or waist to hip ratio (WtHR) in comparison to the healthy population. Currently, body impedance analysis (BIA), which enables algorithm-based estimation of adipose and lean body mass has been increasingly used. While these methods are convenient and accessible in clinical routine practice, their accuracy in reflecting malnutrition and capability to differentiate visceral adipose tissue (VAT) and subcutaneous adipose tissue (SAT) compartments are limited (5–7). Anthropometric measurements tend to underestimate the incidence of obesity and malnutrition, especially with the coexistence of disease both during the initial assessment and over the longer-term following the treatment (8, 9). BIA is safe and demonstrates higher sensitivity than anthropometric methods, but underestimates the amount of adipose tissue in lean children and overestimates in obese ones (5). Although there are imaging methods including dual-energy x-ray absorptiometry (DXA) and computed tomography (CT) which directly discern body compartments with high accuracy, their role in the pediatric population is limited due to the radiation burden (10–12). Another diagnostic tool frequently used in children is magnetic resonance imaging (MRI). Due to the different magnetic properties of water and fat-bound protons, this radiation-free technique allows to assess lean and adipose tissue compartments (13–17). However, a dedicated MRI whole-body protocol for the assessment of nutritional status is highly costly and time-demanding thus is limited in clinical use. In this context, it seems crucial to establish a simple and fast method of VAT and SAT quantification using MRI which can be obtained during the regular diagnostic protocol. A method that meets these requirements was already validated in adult population fat quantification from a single CT and MRI slice at the L2-L3 vertebral level (18–22). With this approach, all adipose tissue measurements can be obtained from routine diagnostic protocol with high correlation to MRI whole-body examination adipose tissue volumes.

Considering the limitation of currently available methods, this study aimed to establish the gender-dependent reference normative values of MRI-derived visceral and subcutaneous adipose tissue in a healthy pediatric population, which can serve as reference standards in the evaluation of body composition in children and adolescence with nutrition disorders.

## Materials and Methods

### Patients

This retrospective study was approved by the Institutional Ethics Committee the approved number of our project is NKBBN/443/2018. Eligible participants were children and adolescence aged 0–18 who underwent MRI examination of the abdomen or pelvis in the years 2010–2020. The local database was searched by use of the dedicated search engine MedStream Designer (MSD) and 1,315 records were found. Exclusion criteria included incorrect search by MSD (281), examinations without T2-weighted sequences (48), T2-weighted sequences distorted by artifacts (47), a history of oncological or hematological disease, hydronephrosis, ascites, glycogen storage diseases (520), patients post nephrectomy, or other surgical procedure (59). The remaining 24 MRI records were follow up studies thus were excluded from the analysis (23). The MRI examinations of children aged 0–5 were also excluded due to insufficient sample size (74). The final analysis included a total of 262 children or adolescence aged 6–18 years (111 girls, and 151 boys) without changes or with changes of benign origin.

### Demographic Characteristics

Demographic characteristics included patients' age, weight, and height at time of MRI examination. BMI was calculated for each subject by dividing weight in kilograms by square of the height in meters.

### Imaging Method

Three different MRI systems were used: two 1.5T systems Magnetom Aera and Magnetom Sola (Siemens Healthineers, Erlangen, Germany) and one 3.0T system Philips Achieva 3.0 TX (Philips Medical Systems Nederlands, Best, Netherlands). MRI examinations of the abdomen and/or pelvis were performed by using the standard protocols. A standard TSE T2-weighted sequence in the transverse plane was taken for analysis. A single slice at the level of the second lumbar vertebra was selected for visceral and subcutaneous adipose tissue evaluation.

### Adipose Tissue Quantification

The fat tissue compartment was segmented into SAT and VAT. The SAT was defined as subcutaneous fat externally of the abdominal and back muscles. The VAT was defined as adipose tissue inside of the abdominal cavity, excluding fat depots within abdominal and back muscles and fat tissue extending beyond the posterior outline of the vertebral body. Both arms visible on the analysis page were excluded from adipose tissue quantification.

**Figure 1:**
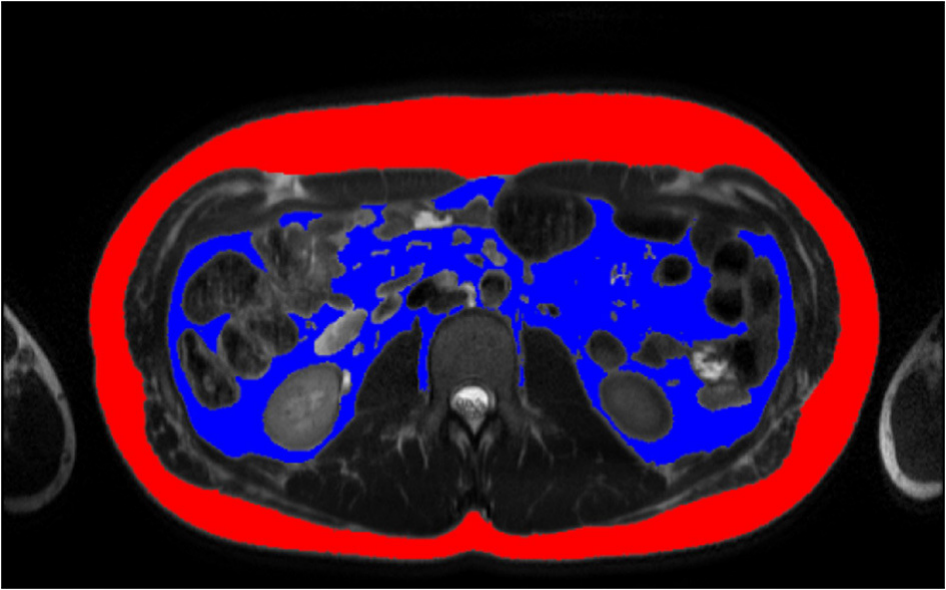
Example of tissue SAT (red) and VAT (blue) segmentation of 14 years old boy, BMI: 27.7 kg/m^2^ by MP. BMI, Body Mass Index, MP, second radiologist; SAT, subcutaneous adipose tissue; VAT, visceral adipose tissue.

Semi-automatic body composition analysis was performed with the use of parametric Magnetic Resonance Imaging v1.2.31-b (pMRI) software. The program is freeware available at the website www.parametricmri.com. The T2-weighted sequence was loaded into pMRI and processed with the volumetric region of interest analysis module which allows for segmentation and volumetric quantification of adipose tissues. A single slice at the level of the second lumbar vertebra was selected for the assessment of adipose tissue. For the analysis of SAT and VAT signal intensity, thresholds were manually set. After signal intensity-based segmentation, all data sets were visually revised (Figure 1). Misclassified tissues were corrected by two operators. One hundred seventy-two sets by KM (2nd year of specialization in radiology) and ninety sets by MP (radiologist with 15 years of experience in MRI). The average time needed for analysis and correction of a single data set was ~between 5 and 15 min. The example of segmented cross-section is presented in Figure 1.

### Statistical Analysis

#### Statistical Analysis of MRI Images

Agreement of segmentation results between observers and intra-observer reproducibility were assessed by using the Bland-Altman plots. The limits of agreement of the Bland-Altman plots were defined as the mean differences ±90% confidence intervals. Statistical analysis was performed in R version 4.1.0.

#### Statistical Analysis of Percentile Charts

Sex-specific BMI-for-age, SAT-for-age and VAT-for-age percentile curves and z-scores were constructed using the lambda-mu-sigma (LMS) method (24) and LMSChartMaker Light version 2.54 software (23). Identification of outliers was made by inspecting the z-score plot of each variable. None of the outliers were considered to be made due to mistakes of data recording or transferring. Following WHO guidelines (25, 26), derivation of percentiles was enabled only within the interval of z-scores between −3.0 and 3.0. To avoid assumptions about the distribution of data beyond the limits of observed values, the standard deviation at each age beyond this limit was fixed at the distance −2.5 SD and 2.5 SD correspondingly. In boys four SAT, three VAT, and one BMI values were fixed; in girls-only one SAT, two VAT, and one BMI values were fixed.

The LMS is based on the assumption that by use of Box-Cox transformation any anthropometric data such as BMI can be converted to a normal distribution for any given age (age was used as a continuous variable). Natural cubic splines with knots at each distinct age t were fitted to create three smooth curves representing the skewness L(t) [Box-Cox transformation], the median M(t), and the coefficient of variation S(t) of the original data as they vary with age:


$$\begin{array}{l} \text{C}\alpha \left(\text{t}\right)=\text{M}\left(\text{t}\right)\text{x}{\left[1+\text{L}\left(\text{t}\right)\text{xS}\left(\text{t}\right)\text{xZ}\alpha \right]}^{1/\text{L}\left(\text{t}\right)} \end{array}$$

where Zα is the α-quantile of a standard normal distribution and Cα(t) is a percentile corresponding to Zα. Equivalent degrees of freedom (edf) L(t), M(t), and S(t) measure the complexity of each fitted curve. In our limited sample size, for each data set the standard edf of L3, M5, S3 was chosen, as further fitting made no significant improvements to our model (23).

## Results

The inter-observer agreement was assessed based on 15 sets of randomly selected MRI examinations segmented separately by both radiologists (KM and MP) (Supplementary Figures 1, 2). The same set of images was subsequently resegmented by one radiologist (K.M.) for the evaluation of the intra-observer reproducibility. Results in form of Bland-Altman plots are presented in the supplementary material (Supplementary Figures 3, 4). For SAT both intra- and inter-observer mean differences were at the level of 0.07 cm^2^. The actual differences were up to 2 cm^2^ for intra- and 0.5 cm^2^ for inter-observer measurements which represents disagreement at a level of 1% for corresponding measurements. Slightly higher intra- and inter-observer disagreement was noted in VAT segmentation reaching accordingly up to 2.4 cm^2^ (mean −0.04 cm^2^) and 2.7 cm^2^ (mean 0.08 cm^2^). In those cases maximum difference in measurements was around 3%.

**Figure 2:**
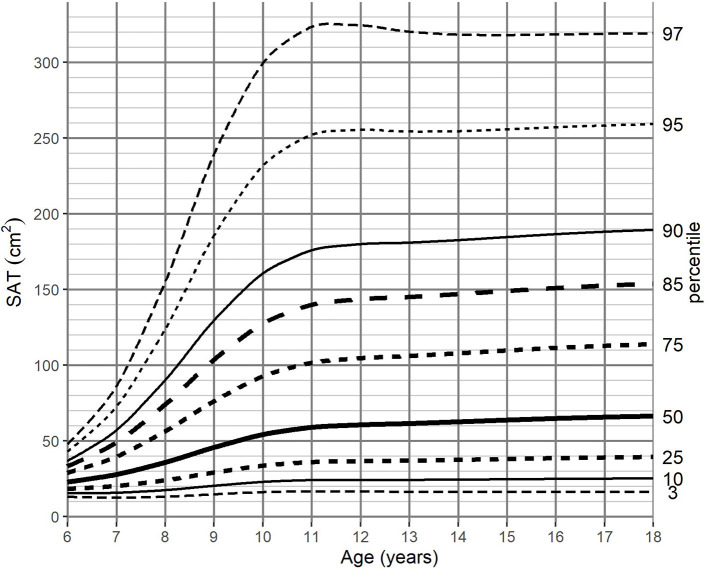
SAT-for-age (cm^2^) percentile charts for boys aged from 6 to 18 years. SAT, subcutaneous adipose tissue.

**Figure 3:**
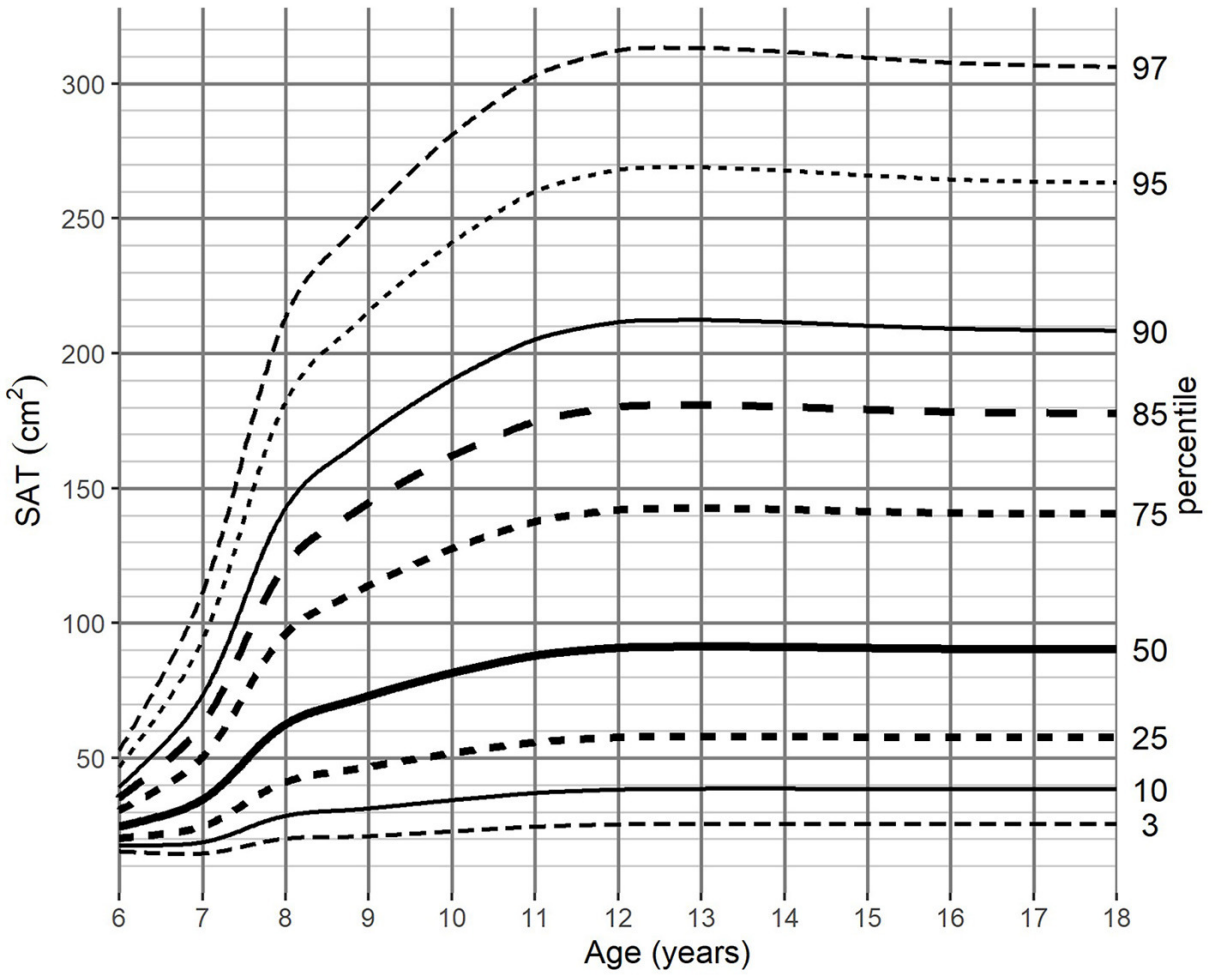
SAT-for-age (cm^2^) percentile charts for girls aged from 6 to 18 years. SAT, subcutaneous adipose tissue.

**Figure 4:**
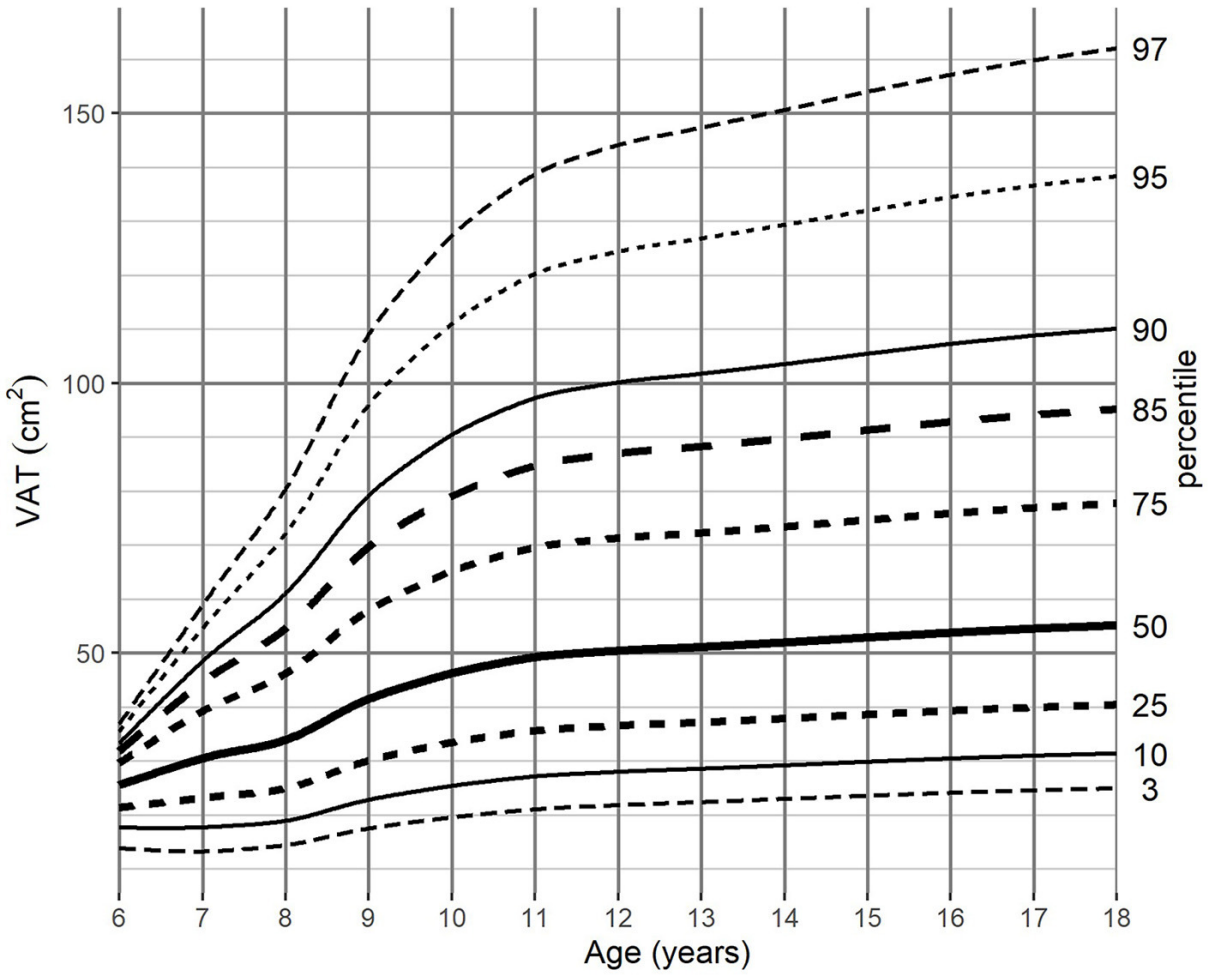
VAT-for-age (cm^2^) percentile charts for boys aged from 6 to 18 years. VAT, visceral adipose tissue.

**Figure 5:**
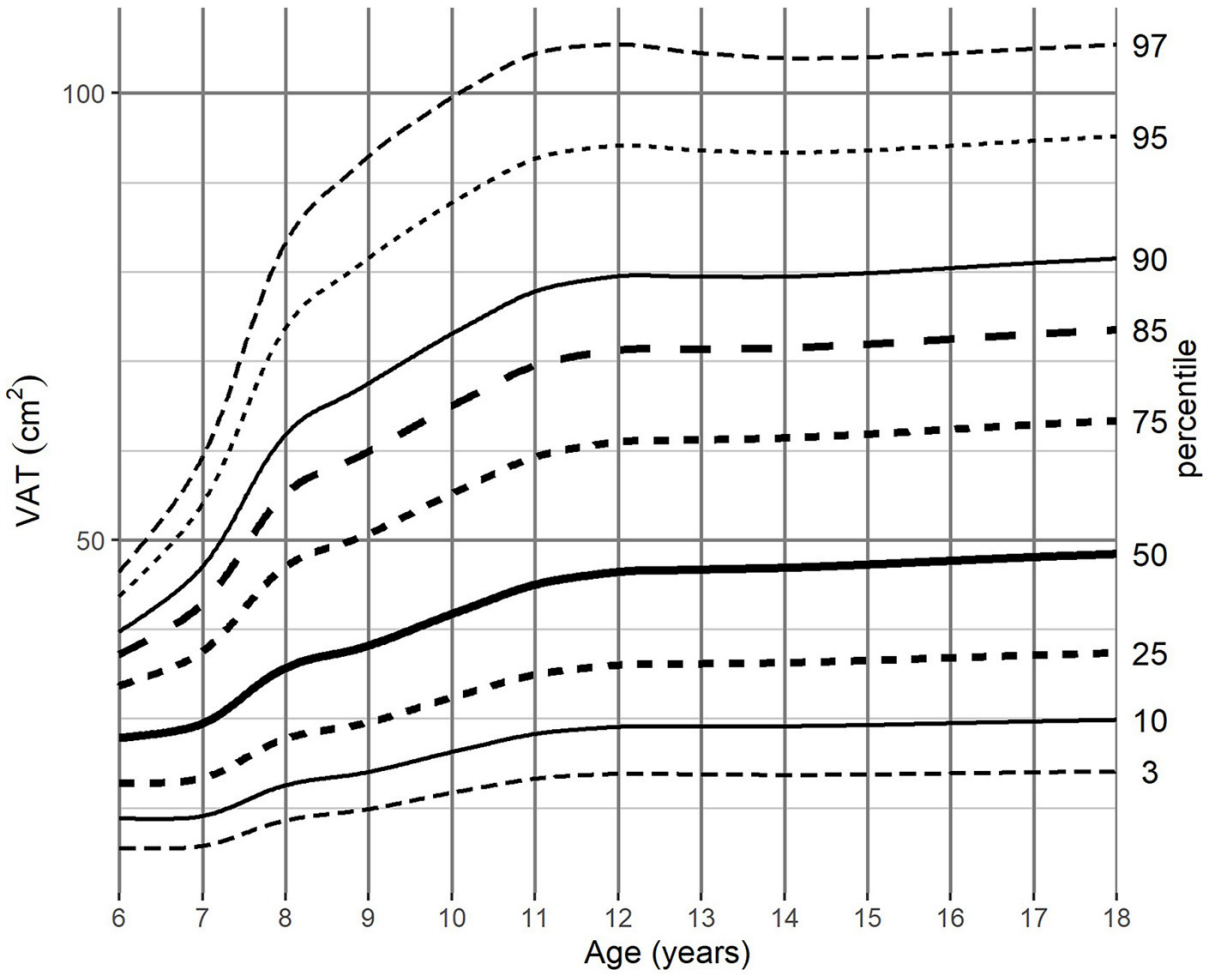
VAT-for-age (cm^2^) percentile charts for girls aged from 6 to 18 years. VAT, visceral adipose tissue.

For the adjustment of BMI-for-age, SAT-for-age, and VAT-for-age percentiles the 262 MRI pediatric examinations (111 girls, and 151 boys) aged 6–18 (mean age of 12.49 years) were enrolled. The SAT and VAT reference values in each age group for boys and girls are presented in Tables 1–**4**. Based on the results percentile curves for SAT and VAT were calculated and presented in Figures 2–5.

**Table 1:** SAT-for-age (cm^2^) references for boys.

**Age** ** (years)**	**-2 SD**	**-1SD**	**1 SD**	**2 SD**	**P3**	**P5**	**P10**	**P25**	**P50**	**P75**	**P85**	**P90**	**P95**	**P97**
6	12.59	16.65	32.90	50.62	13.00	13.86	15.34	18.37	22.84	29.03	33.38	36.86	43.04	47.88
7	11.93	17.64	47.90	94.22	12.47	13.63	15.71	20.29	27.80	39.65	48.96	57.03	72.69	86.13
8	12.37	20.17	71.96	174.74	13.07	14.60	17.45	24.06	35.83	56.40	74.04	90.25	123.97	155.09
9	13.82	23.94	100.41	274.09	14.69	16.64	20.33	29.19	45.72	76.26	103.69	129.70	185.76	239.42
10	15.10	27.26	123.36	342.98	16.13	18.45	22.89	33.69	54.22	92.71	127.52	160.58	231.70	299.44
11	15.53	28.92	135.35	368.56	16.66	19.20	24.08	36.05	58.90	101.63	139.91	175.90	252.16	323.41
12	15.35	29.27	139.05	367.50	16.52	19.15	24.23	36.72	60.54	104.67	143.67	179.89	255.33	324.37
13	15.12	29.39	140.52	360.87	16.31	19.00	24.21	37.04	61.42	106.13	145.11	180.88	254.24	320.19
14	15.04	29.69	142.40	357.15	16.26	19.02	24.37	37.55	62.53	107.89	146.98	182.51	254.45	318.21
15	15.04	30.07	144.48	355.74	16.29	19.12	24.61	38.13	63.70	109.76	149.07	184.53	255.64	317.96
16	15.06	30.43	146.40	355.33	16.34	19.23	24.84	38.67	64.76	111.45	151.01	186.47	257.04	318.37
17	15.08	30.72	147.97	355.13	16.38	19.32	25.03	39.12	65.63	112.84	152.59	188.07	258.23	318.79
18	15.09	30.95	149.23	354.93	16.40	19.38	25.18	39.47	66.33	113.95	153.86	189.33	259.14	319.09

*P, percentile; SAT, subcutaneous adipose tissue; SD, standard deviation*.

Corresponding BMI growth charts are presented in the supplementary material in correlation to age (Supplementary Figures 5, 6). Among both genders, BMI increased continuously during childhood and adolescence, reaching a median of 22.5 kg/m^2^ in boys and 21.7 kg/m^2^ in girls at the end of the observed age range (18 years). In the groups between 8 and 10 years old, the flattening of the centile curves for BMI was observed, especially noticeable in the percentile range from 3 to 50.

The distribution of SAT percentiles were different between both genders. In boys, a continuous increase was observed throughout all age groups, reaching the median of 66.33 cm^2^ at 18 years of age. In girls, at the beginning of maturity-onset (from age 7 to 11 years), a dynamic increase of SAT surface area was noted, which then stabilized at the age of 14 years (median of 91.1 cm^2^).

For SAT, the difference between the 3rd and 97th percentile reached a maximum of 307.85 cm^2^ for boys 12 years of age, while the maximum difference for girls (287.54 cm^2^) was attained at 13 years of age (Tables 1, 2).

**Table 2:** SAT-for-age (cm^2^) references for girls.

**Age** ** (years)**	**-2 SD**	**-1SD**	**1 SD**	**2 SD**	**P3**	**P5**	**P10**	**P25**	**P50**	**P75**	**P85**	**P90**	**P95**	**P97**
6	15.01	18.77	34.93	56.73	15.38	16.18	17.55	20.38	24.64	30.84	35.44	39.33	46.70	52.96
7	13.94	21.32	61.34	121.92	14.62	16.11	18.81	24.79	34.69	50.41	62.75	73.41	93.97	111.47
8	18.75	33.82	118.62	231.60	20.09	23.06	28.58	41.20	62.52	96.01	121.48	142.78	181.99	213.50
9	19.40	37.72	141.25	271.92	21.00	24.57	31.29	46.81	73.12	114.04	144.67	169.91	215.57	251.54
10	20.97	41.60	158.17	303.46	22.77	26.78	34.34	51.86	81.57	127.65	162.00	190.22	241.06	280.94
11	22.57	44.82	170.59	237.12	24.51	28.83	36.99	55.90	87.96	137.68	174.72	205.14	259.92	302.87
12	23.37	46.34	175.94	337.23	25.38	29.84	38.26	57.75	90.80	142.03	180.19	211.54	267.98	312.23
13	23.59	46.67	176.64	338.17	25.61	30.10	38.56	58.14	91.30	142.66	180.90	212.30	268.83	313.15
14	23.64	46.66	176.02	336.58	25.65	30.13	38.57	58.09	91.11	142.22	180.26	211.48	267.68	311.72
15	23.60	46.53	175.01	334.14	25.61	30.07	38.48	57.89	90.72	141.47	179.21	210.18	265.88	309.52
16	23.57	46.43	174.20	332.08	25.57	20.03	38.41	57.75	90.42	140.88	178.37	209.12	264.39	307.67
17	23.59	46.43	173.82	330.93	25.59	30.04	38.41	57.73	90.33	140.64	177.98	208.59	263.60	306.65
18	23.63	46.47	173.69	330.31	25.63	30.08	38.46	57.77	90.35	140.57	177.84	208.37	263.22	306.12

*P, percentile; SAT, subcutaneous adipose tissue; SD, standard deviation*.

The distribution of VAT percentiles was comparable for both genders. Both boys and girls showed a continuous increase in surface areas in all age groups, reaching the median of 55.08 cm^2^ in boys and 48.41 cm^2^ in girls at 18 years of age, respectively.

The difference of VAT areas between extreme percentiles increased continuously until age of 12 years in girls and until the end of the observed age range in boys. At this age, the difference of 81.15 and 137.14 cm^2^, respectively, was attained, however in girls from 11 years onwards no substantial differences were noted (Tables 3, 4).

**Table 3:** VAT-for-age (cm^2^) references for boys.

**Age** ** (years)**	**-2 SD**	**-1SD**	**1 SD**	**2 SD**	**P3**	**P5**	**P10**	**P25**	**P50**	**P75**	**P85**	**P90**	**P95**	**P97**
6	13.00	19.35	31.61	37.60	13.77	15.28	17.58	21.38	25.54	29.65	31.38	33.31	35.48	36.89
7	12.38	20.06	43.99	61.09	13.16	14.82	17.64	23.13	30.46	39.22	44.55	48.42	54.58	58.85
8	13.60	21.46	53.64	84.95	14.36	15.99	18.87	24.91	33.91	46.20	54.55	61.05	72.15	80.42
9	16.56	25.87	68.35	116.36	17.44	19.35	22.76	30.07	41.46	57.89	69.64	79.12	95.95	109.01
10	18.53	28.76	77.50	136.57	19.50	21.58	25.33	33.42	46.23	65.17	79.04	90.41	110.97	127.27
11	19.97	30.68	82.99	149.33	20.97	23.16	27.08	35.59	49.18	69.55	84.69	97.22	120.21	138.69
12	20.74	31.57	85.23	155.52	21.76	23.96	27.93	36.55	50.37	71.29	86.99	100.10	124.36	144.08
13	21.29	32.16	86.46	159.30	22.31	24.52	28.50	37.16	51.07	72.25	88.26	101.70	126.77	147.32
14	21.87	32.82	87.88	163.08	22.90	25.13	29.13	37.86	51.91	73.40	89.72	103.49	129.30	150.60
15	22.45	33.52	89.44	166.92	23.49	25.74	29.79	38.61	52.84	74.67	91.32	105.41	131.95	153.97
16	22.98	34.17	90.91	170.48	24.03	26.31	20.40	39.32	53.72	75.87	92.83	107.22	134.44	157.11
17	23.43	34.72	92.17	173.53	24.49	26.79	30.92	39.92	54.47	76.90	94.12	108.77	136.56	159.79
18	23.80	35.18	93.21	176.05	24.87	27.19	31.35	40.42	55.08	77.75	95.18	110.04	138.30	162.01

*P, percentile; SD, standard deviation; VAT, visceral adipose tissue*.

**Table 4:** VAT-for-age (cm^2^) references for girls.

**Age** ** (years)**	**-2 SD**	**-1SD**	**1 SD**	**2 SD**	**P3**	**P5**	**P10**	**P25**	**P50**	**P75**	**P85**	**P90**	**P95**	**P97**
6	14.83	20.56	36.84	47.88	15.44	16.70	18.80	22.75	27.82	33.69	37.20	39.73	43.71	46.45
7	15.15	20.94	42.31	62.16	15.74	16.97	19.08	23.35	29.46	37.52	42.89	47.03	54.07	59.29
8	17.92	24.76	54.25	88.73	18.59	20.02	22.52	27.75	35.68	47.01	55.15	61.79	73.77	83.29
9	19.15	26.40	58.89	99.56	19.85	21.36	24.01	29.59	38.18	50.71	59.91	67.53	81.56	92.95
10	20.90	28.88	63.92	106.31	21.68	23.34	26.26	32.37	41.71	55.21	65.01	73.07	87.74	99.52
11	22.40	31.12	68.38	111.08	23.25	25.07	28.27	34.92	44.98	59.29	69.51	77.80	92.67	104.40
12	22.89	32.03	70.12	111.84	23.79	25.70	29.05	35.99	46.39	60.98	71.25	79.49	94.09	105.44
13	22.81	32.13	70.24	110.56	23.73	25.68	29.09	36.15	46.65	61.21	71.35	79.42	93.58	104.47
14	22.73	32.19	70.38	109.79	23.67	25.65	29.11	36.26	46.85	61.41	71.48	79.44	93.31	103.89
15	22.77	32.38	70.81	109.79	23.72	25.73	29.26	36.50	47.21	61.85	71.91	79.83	93.57	104.00
16	22.87	32.63	71.38	110.18	23.83	25.88	29.46	36.81	47.64	62.38	72.48	80.40	94.09	104.44
17	22.98	32.88	71.93	110.66	23.96	26.04	29.66	37.11	48.05	62.90	73.03	80.97	94.64	104.95
18	23.08	33.09	72.42	111.09	24.07	26.17	29.84	37.37	48.41	63.35	73.52	81.47	95.13	105.42

*P, percentile; SD, standard deviation; VAT, visceral adipose tissue*.

## Discussion

This the first study which demonstrates the reference values of the subcutaneous and visceral adipose tissue as the percentile charts for girls and boys from 6 to 18 years of age.

Currently, the importance of adequate nutritional status during illness is strongly emphasized. Over the years a wide variety of VAT metabolic activity was confirmed highlighting the importance of the body composition assessment during treatment (27–29). Volume and distribution of adipose tissues determinate the type and intensity of malnutrition and therefore enable adequate nutritional support (30, 31).

Appropriate assessment of VAT in the pediatric population is considered to be a serious problem. Most of currently available measurement methods have limitations as discussed in the introduction (8, 9). In contrast, MRI enables direct, accurate, quantitative assessment of all compartments of body fat and is a radiation-free technique which allows safe and long-term observation in body composition changes during growth when compared to CT.

Our study plan was to use the safest method with high efficiency in the quantitative assessment of VAT and SAT. This can be done with MRI imaging which is a commonly used technique in pediatric population during the routine diagnostic process. The accuracy and reproducibility of the MRI examination in the assessment of adipose tissue have already been proved in both adult and pediatric patients (14, 32). The semi-automatic methodology used in our study is consistent with previous studies. In our study, SAT and VAT surface area results obtained by both radiologists on slices at the level of second lumbar vertebrae of randomly selected patients showed high intra-observer reproducibility and inter-observer agreement (Supplementary Figures 1–4). Both in SAT and VAT plots, the mean difference between radiologists was insignificant up to 2.5 cm^2^ indicating that one of them selected larger areas as adipose tissue. In both intra- and inter-observer Bland-Altman plots, greater differences between measurements were noted in VAT groups. However, actual differences in measured adipose tissue areas were up to 2.5 cm^2^, which makes this difference almost negligible. The high correlation between observers obtained in our study indicates the reliability of SAT and VAT measurements suggesting that these findings can be used to build models of the percentile charts.

Considering the purpose of our study and pediatric population, we had to change the current MRI image sequence approach which is commonly used for adipose tissue quantification. To date, the majority of published studies have used T1-weighted water-fat sequences (called Dixon sequence). While these sequences have a short acquisition time, the quality of acquired images are strongly dependent on the ability to breath-hold during the examination. The sufficiently long breath-hold is difficult for young children and impossible in case of sedation. Thus, Dixon images of abdomen and pelvis acquired in children are frequently burdened by movement artifacts, making this impossible to evaluate the change in body composition (33). To overcome this limitation, our study used T2-weighted sequences. In the study of Pescatori et al. has shown that the sensitivity and specificity of T1- and T2-weighted sequences in the assessment of adipose tissue are comparable but the results of T2-weighted sequences tended to be more reproducible (32). Furthermore, T2-weighted sequences are included in all standard examination protocols of the abdominal and/or pelvis cavity. Thus, utilizing these sequences for the assessment of SAT and VAT has no major impact on examination and sedation time.

Proper assessment of obtained images requires the involvement of highly qualified personnel. Although tools for manual or semi-automatic SAT and VAT quantification are widely available, segmentation throughout all slices at the level of abdominal or pelvis cavities is time-consuming and impractical (34). Therefore, the quantity of particular adipose tissue depots is usually estimated based on a single cross-section image (18–22, 35, 36). According to the current knowledge, in children cross-sections at the height of L2 vertebrae are the most accurate and correlate to the total amount of SAT and VAT (35, 36). Although in the future artificial intelligence (AI) algorithms may simplify the adipose tissue segmentation process, the current utilization of the single-slice approach is the most optimal solution.

In this context, in the present study by creating the SAT and VAT percentile charts we provide a tool that can be widely and easily implemented in clinical practice. The percentile charts are costless, easy, quick to apply, and enable observation of the growth tendencies over the longer term. The most used percentile charts in pediatric populations are weight, height, and BMI charts (37). However, BMI percentile curves are created by averaging not only SAT and VAT, but also muscle and internal organs mass. As a result, the BMI percentile charts cannot properly illustrate changes in the adipose tissue during children's growth (6, 38). Regardless of gender, the BMI values presented a continuous increase from 6 to 18 years of age both in data presented by WHO (39), as well as in our study. However, only the value of VAT showed a similar upward trend. The SAT surface area stabilized around the age of 12 for both boys and girls. The distribution of BMI standard deviations scores in our population was similar to the regional reference values (40). However, flattening of the BMI curves in the age range from 8 to 10 years in both sexes was noticeable which may be related to the size of our study group.

It should be emphasized that in the same age range, in the contrary to BMI, the SAT, and VAT percentile curves showed a continuous increase. These findings may indicate that our method is more sensitive and precise at reflecting the actual changes in the amount and distribution of body fat.

In our study, data from children with known disorders affecting growth were excluded. The presented standard deviation scores and percentiles should be considered as growth references (not growth standards according to the WHO terminology) because we did not identify environmental conditions “likely to favor the achievement of children's full genetic growth potential” (25). To better monitor, the growing problem of overweight and obesity among children and adolescents in the recommendations of the pediatric obesity experts committee the cut-off values have been determined at the level of 85th and 95th percentiles as the best equivalents of adults' 25th and 30th BMI values (41). Similarly, in our study for the SAT and VAT percentile charts we proposed the 85th and 95th percentile curves as warning points, above which attention for overweight is required. Determining the exact percentile cut-off for SAT and VAT overweight and obesity requires further research on a larger population.

This study has several limitations. Firstly, the number of participants was relatively small, as percentile charts are usually created during population-based prospective studies. Our study was conducted at a single-center, therefore our results only refer to the Caucasian population. Additionally, semi-automatic adipose tissue assessment is time-consuming and further research on a larger study group would require the implementation of fully automatic tools based on AI deep learning algorithms. Further limitation of this study is the lack of centile charts for children from birth to 5 years of age. Since percentile charts for the youngest children are commonly presented in monthly intervals, our study did not include a sufficient number of healthy participants in these age groups to obtain reliable results.

In conclusion, for the first time, we have shown reference values of SAT and VAT in form of percentile charts for boys and girls during childhood and adolescence. Frequent utilization of MRI examinations in the pediatric population may enable the implementation of our method in clinical practice for body composition assessment and proper nutritional support. In the view of the rapid development of AI deep learning algorithms, there seems to be a high possibility of automatization and incorporation of MRI-based adipose tissue assessment into standard diagnostic protocols.

## Data Availability Statement

The datasets presented in this article are not readily available because the need of agreement from University Clinical Center of Gdańsk. Requests to access the datasets should be directed to mleszczynska@uck.gda.pl.

## Ethics Statement

The studies involving human participants were reviewed and approved by Independent Bioethics Committee for Scientific Research at Medical University of Gdańsk. Written informed consent to participate in this study was provided by the participants' legal guardian/next of kin.

## Author Contributions

KM and MP contributed to the conception or design of the work and drafted the manuscript. KM, WB, MG, MK, PB, DK, and MP contributed to the acquisition, analysis, or interpretation of data for the work. DS, WB, DK, and MG critically revised the manuscript. All authors gave final approval and agree to be accountable for all aspects of work ensuring integrity and accuracy.

## Conflict of Interest

The authors declare that the research was conducted in the absence of any commercial or financial relationships that could be construed as a potential conflict of interest.

## Publisher's Note

All claims expressed in this article are solely those of the authors and do not necessarily represent those of their affiliated organizations, or those of the publisher, the editors and the reviewers. Any product that may be evaluated in this article, or claim that may be made by its manufacturer, is not guaranteed or endorsed by the publisher.
