# Supplementary material for: MRI-Derived Subcutaneous and Visceral Adipose Tissue Reference Values for Children Aged 6 to Under 18 Years
Source: Front Nutr. 2021 Oct 1;8:757274. doi: 10.3389/fnut.2021.757274 (PMC8517194; doi:10.3389/fnut.2021.757274)

Supplementary Material

# Supplementary Figures and Tables

## Supplementary Figures

**Supplementary Figure 1.** Bland-Altman plot of the difference in interobserver SAT segmentation (cm^2^) against the mean SAT segmentation (cm^2^). KM, first radiologist; MP, second radiologist; SAT, subcutaneous adipose tissue; SD, standard deviation.


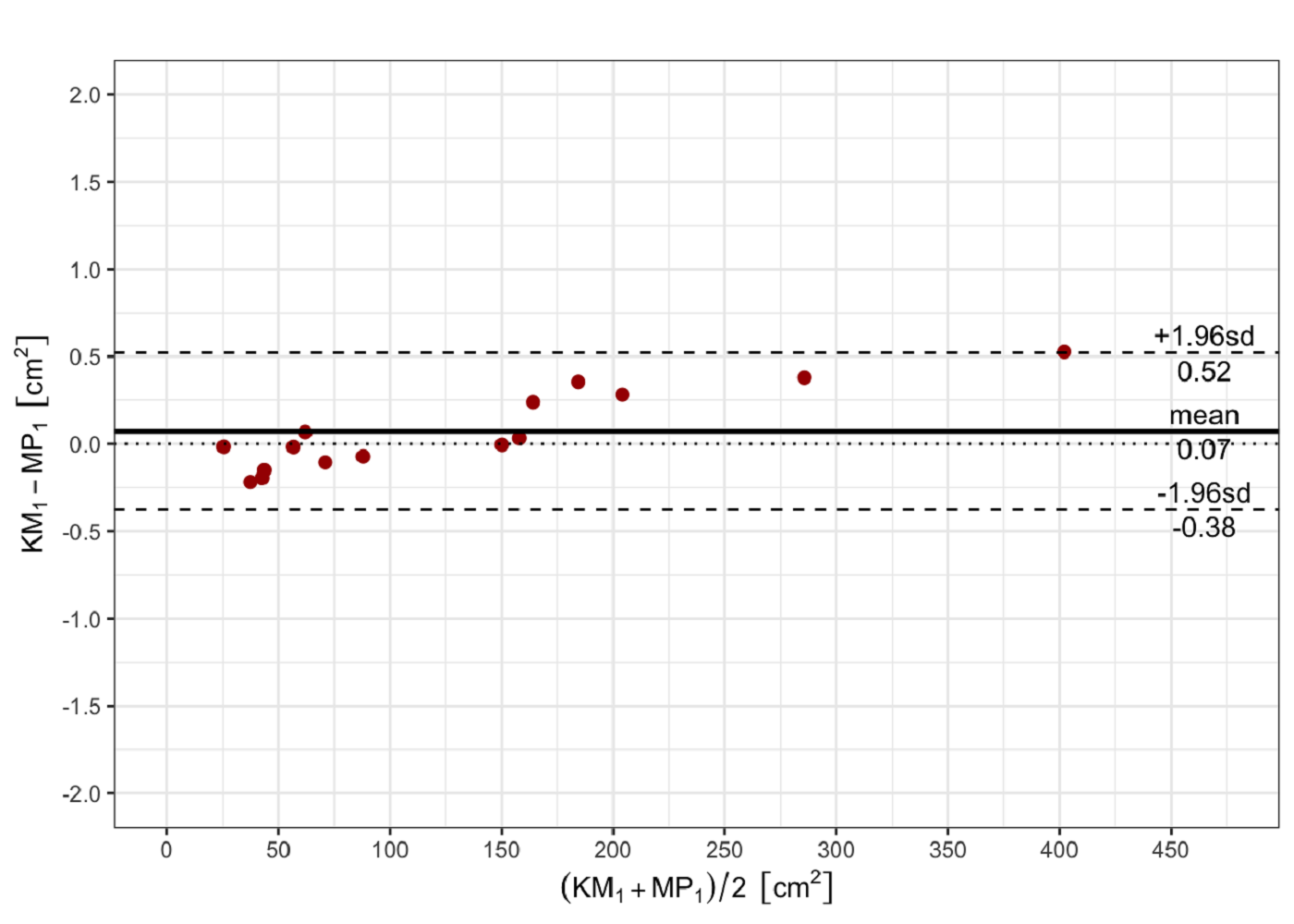


**Supplementary Figure 2.** Bland-Altman plot of the difference in interobserver VAT segmentation (cm^2^) against the mean VAT segmentation (cm^2^). KM, first radiologist; MP, second radiologist; SD, standard deviation; VAT, visceral adipose tissue.


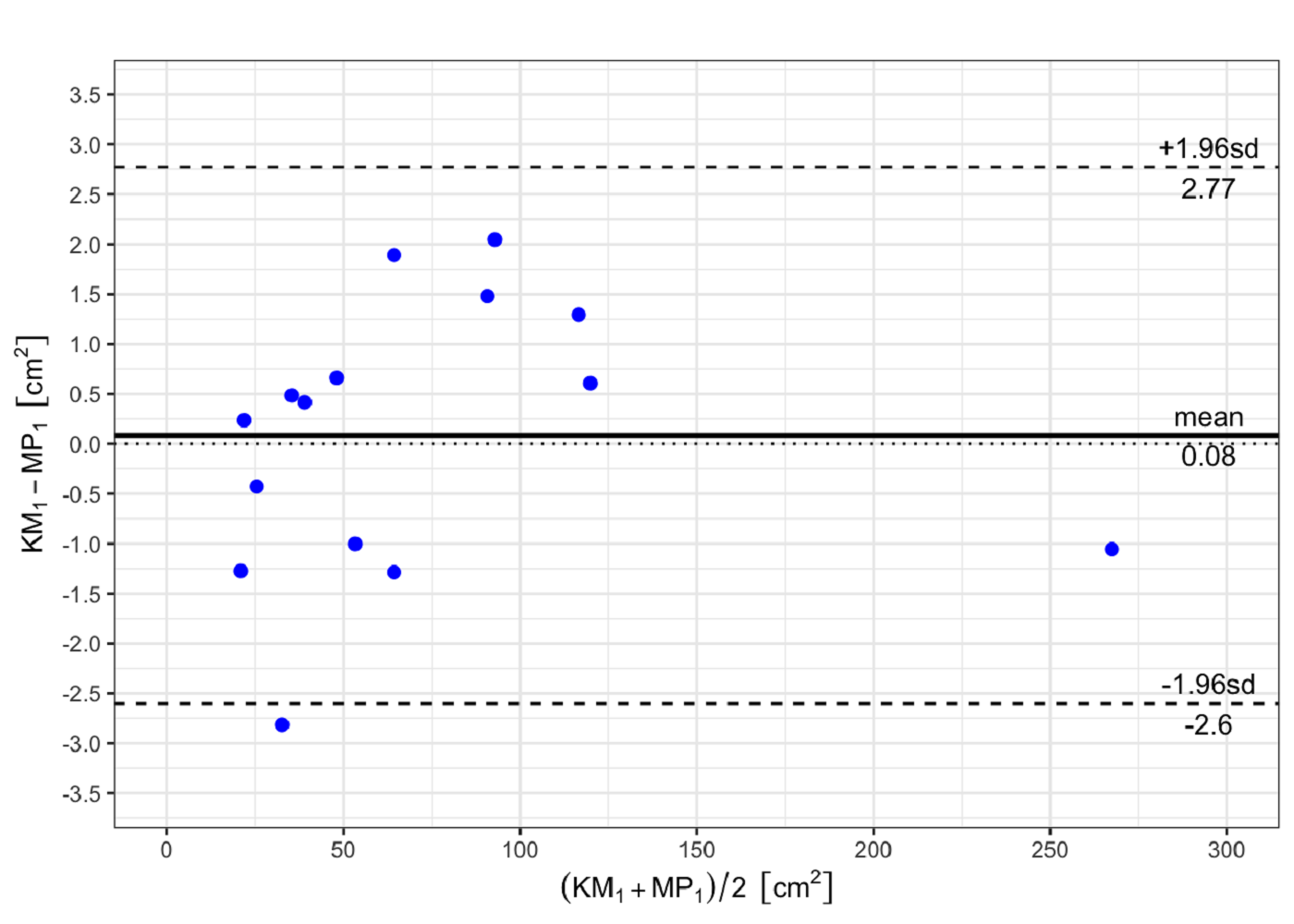


**Supplementary Figure 3.** Bland-Altman plot of the difference in intraobserver SAT segmentation (cm^2^) against the mean SAT segmentation (cm^2^). KM_1_, first radiologist, first measurement; KM_2_, first radiologist, second measurement; SAT, subcutaneous adipose tissue; SD, standard deviation.


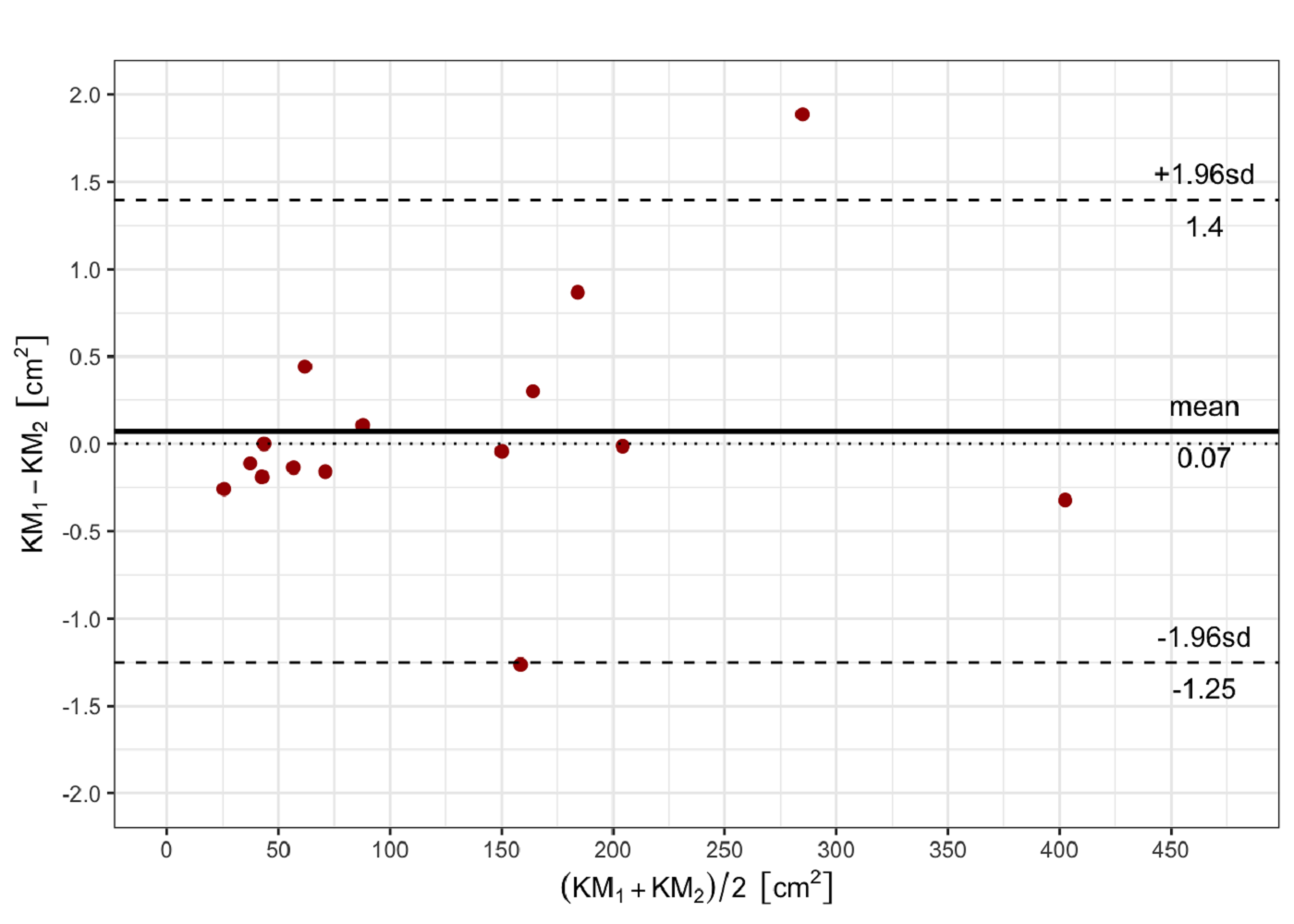


**Supplementary Figure 4.** Bland-Altman plot of the difference in intraobserver VAT segmentation (cm^2^) against the mean VAT segmentation (cm^2^). KM_1_, first radiologist, first measurement; KM_2_, first radiologist, second measurement; SD, standard deviation; VAT, visceral adipose tissue.


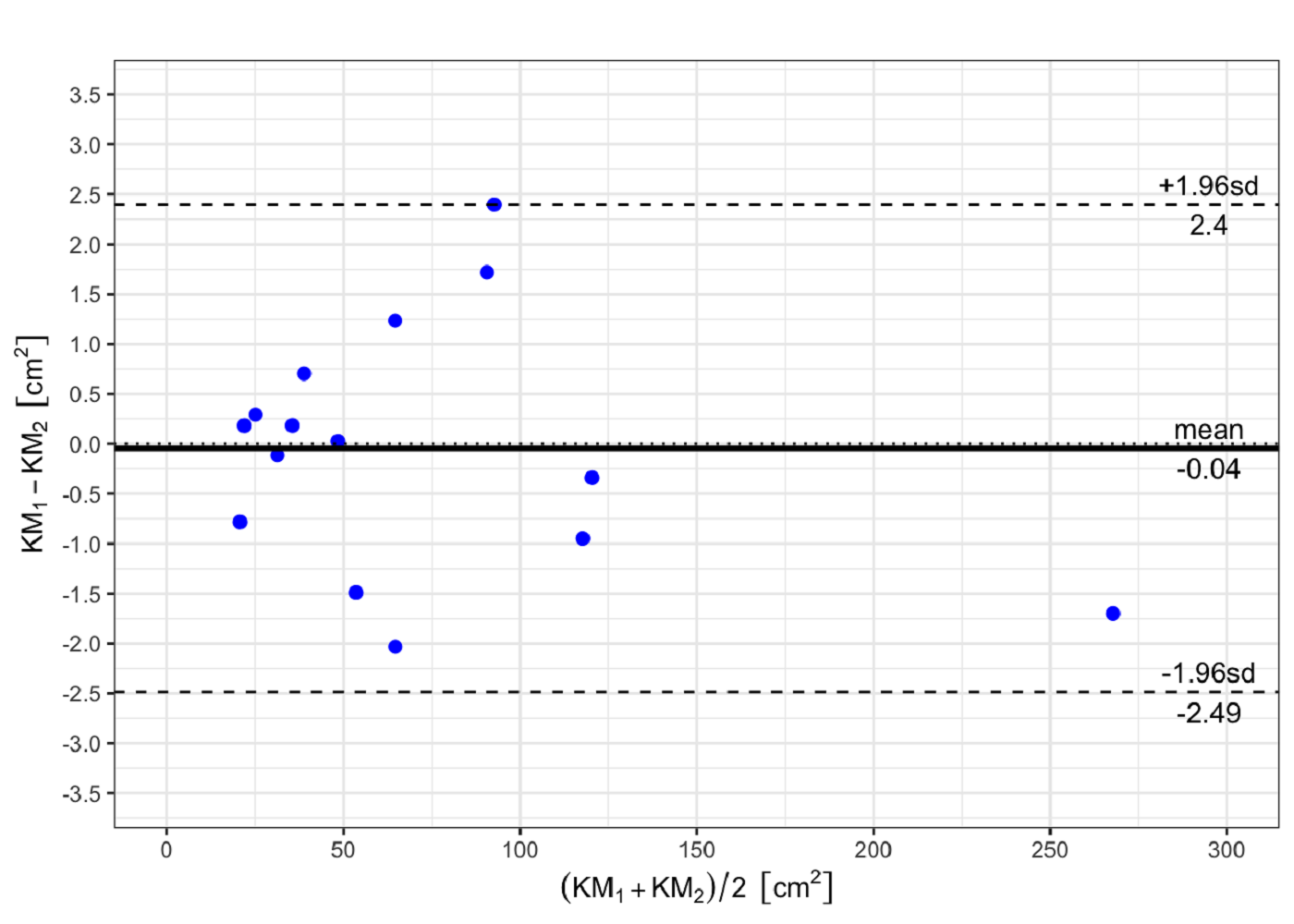


**Supplementary Figure 5.** BMI-for-age (kg/m^2^) percentile charts for boys aged from 6 to 18 years. BMI, Body Mass Index.


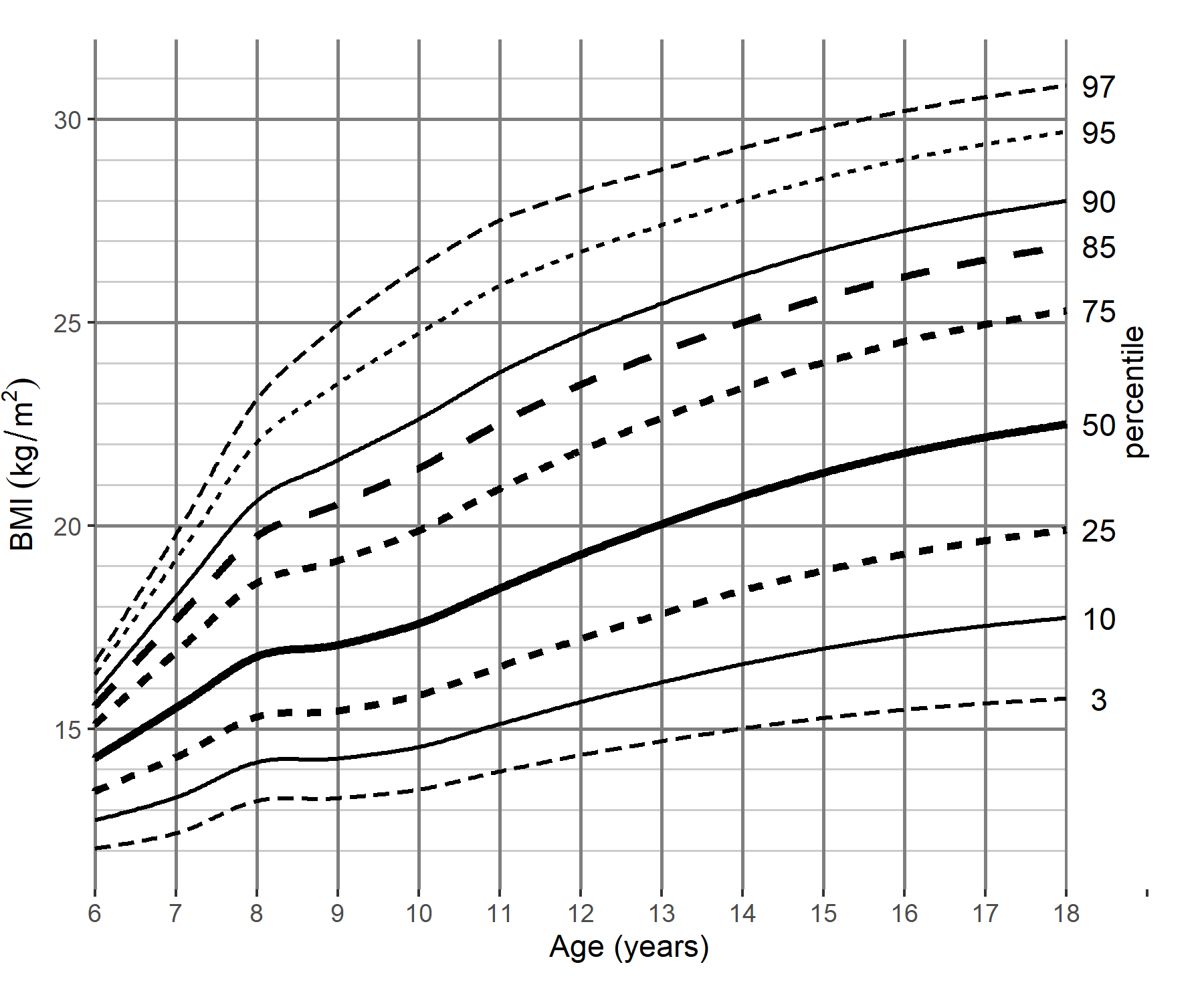


**Supplementary Figure 6.** BMI-for-age (kg/m^2^) percentile charts for girls aged from 6 to 18 years. BMI, Body Mass Index.


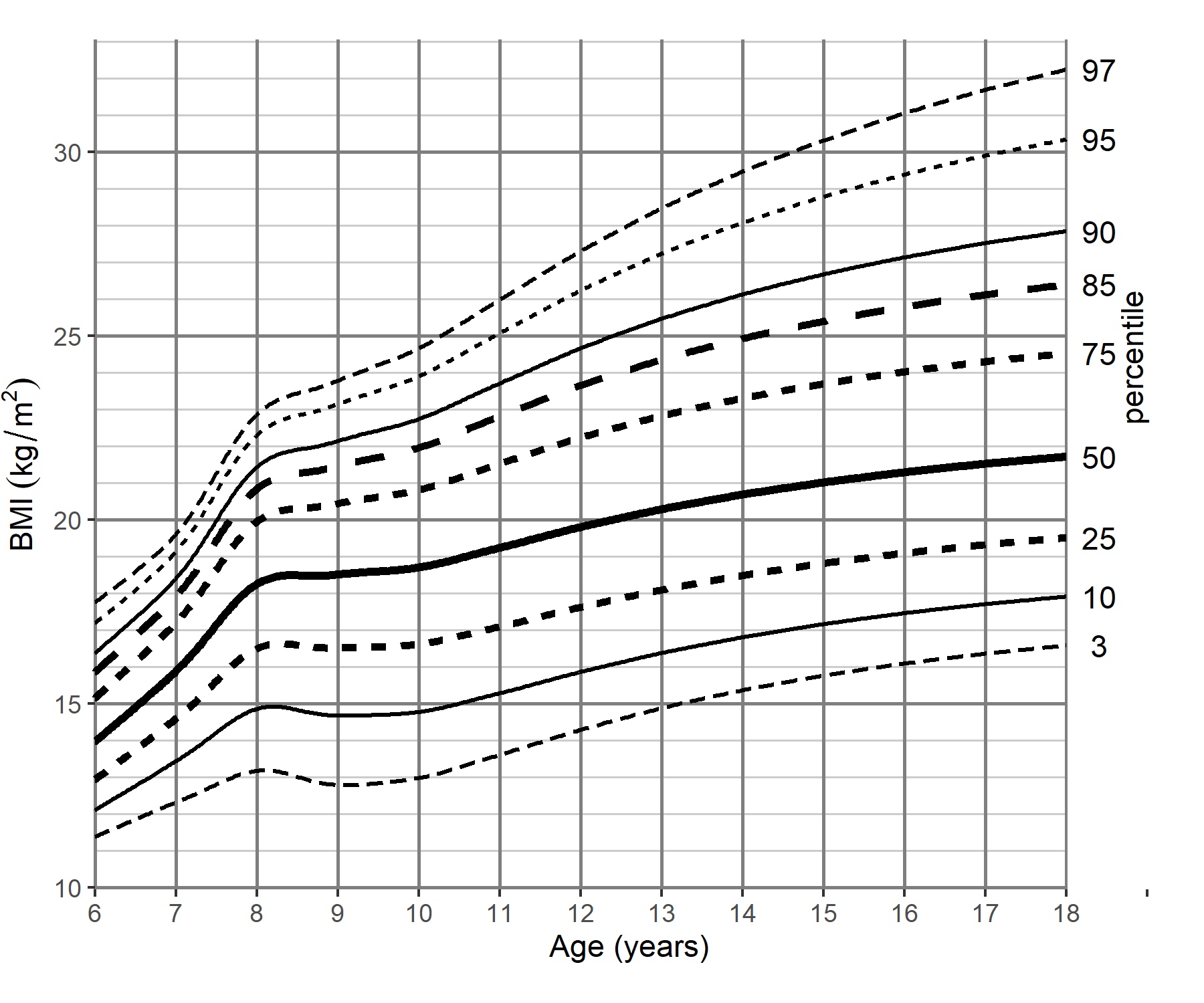

Supplement: Supplementary file 1 [file Data_Sheet_1.docx]
